# Primary Hepatic Epithelioid Hemangioendothelioma Masquerading as a Hepatic Abscess With Infective Picture: A Case Report

**DOI:** 10.7759/cureus.22859

**Published:** 2022-03-05

**Authors:** Jouhar J Kolleri, Ali Khaliq, Sushila B Ladumor, Abiel Berhe Habtezghi, Shieja M Koshy, Mahir Petkar

**Affiliations:** 1 Department of Clinical Imaging, Hamad Medical Corporation, Doha, QAT; 2 Department of Anesthesia, Hamad Medical Corporation, Doha, QAT; 3 Radiology, Orotta National Referral Hospital, Asmara, ERI; 4 Department of Laboratory Medicine and Pathology, Hamad Medical Corporation, Doha, QAT

**Keywords:** positron emission tomography computed tomography, magnetic resonance imaging, computed tomography, hemangioendothelioma, primary hepatic eptheliod hemangioendothelioma

## Abstract

Hepatic endotheiloid hemangioendothelioma (EHE) commonly presents with multilobar involvement with locally aggressive behavior. In most cases, it presents with right upper quadrant abdominal pain, hepatomegaly, and weight loss with metastasis commonly to the lung. We present a 33-year-old woman with hepatic EHE with an initial presentation mimicking hepatic abscess and imaging findings misleading for metastatic liver lesions. It was confirmed on pathology with immunohistochemistry, but the patient could not survive due to her late presentation and the presence of metastatic lesions in the lung.

## Introduction

Epithelioid hemangioendothelioma (EHE) is an unusual endothelial or preendothelial cell vascular tumor formed by epithelioid and histiocyte appearing cells in a mucoid to fibrotic stroma [1]. It has been reported with an incidence of fewer than two cases per 1 million [2]. In the 2020 World Health classification of soft tissue tumors, epithelioid hemangioendothelioma was classified as a malignant vascular tumor because of its 15% chance of metastasis, especially when located in the lungs and the pleura [3]. Patients might present with symptoms such as weight loss, liver enlargement, and pain in the right upper quadrant of the abdomen [4]. The diagnosis of hepatic EHE is confirmed with pathological examination with immunohistochemistry. We present a rare hepatic epithelioid hemangioendothelioma in a female patient which initially resembled a hepatic abscess.

## Case presentation

A 33-year-old woman who was previously healthy was admitted to the hospital with complaints of cough, fever, chills, and right-sided abdominal pain for three months. The abdominal pain was not related to food intake. No change in stool color and no change in bowel habits were noted. The patient denied any similar episodes in the past. Vitals were as follows: temperature: 37.3 °C, heart rate:100 beats per minute, respiratory rate: 16 breaths per minute, and blood pressure: 112/70 mmHg. On general examination, the patient was conscious and oriented. Abdomen examination elicited right upper quadrant tenderness. Other systemic examinations were within normal limits. Laboratory results showed high inflammatory markers, eosinophilia, microcytic anemia, and elevated liver function tests.

**Figure 1:**
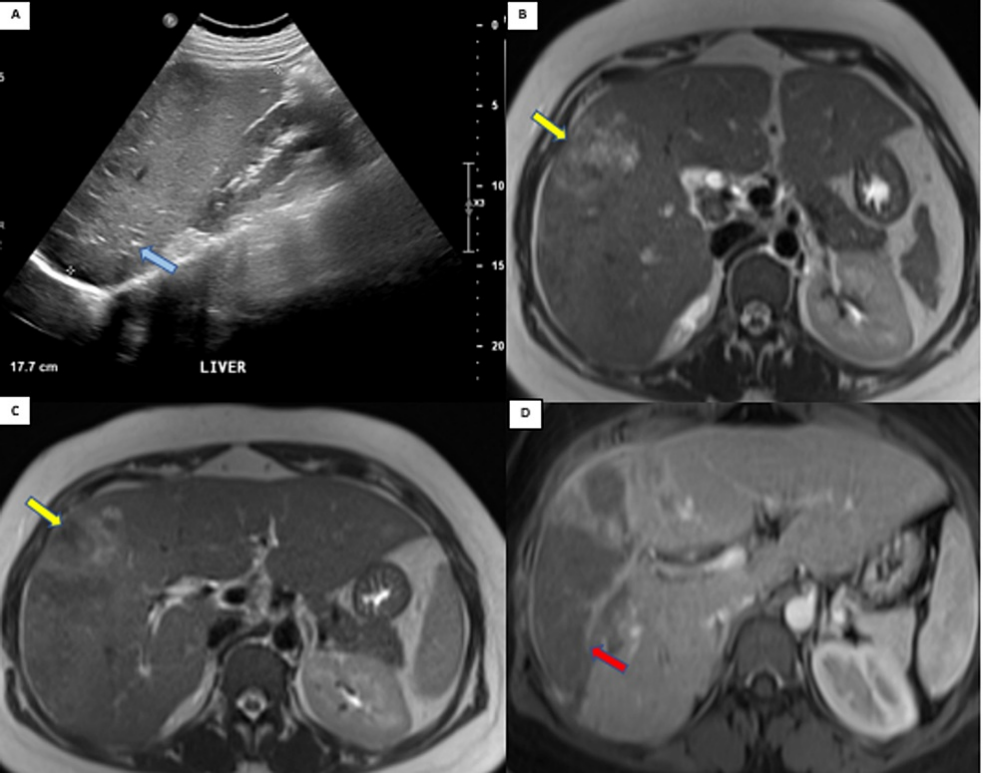
(A) Ultrasound abdomen showing enlarged liver with multiple calcifications (blue arrow). (B and C) MRI T2 haste showing large space-occupying lesion with heterogenous intermediate signal in segment 5 (yellow arrows) and (D) MRI T1 postcontrast showing enlarged liver with heterogeneous parenchymal enhancement and large non-enhancing lesion with septal enhancement (red arrow) in right lobe with multiple surrounding smaller ill-defined lesions.

A chest radiograph showed no evidence of clear infiltration. An ultrasound abdomen was done, which showed an enlarged liver with multiple calcifications. An MRI abdomen was done which showed an enlarged liver and the right lobe demonstrated heterogeneous bright T2 weighted signal intensity with mild diffusion restriction associated with heterogeneous parenchymal enhancement on the postcontrast sequences. There were multiple space-occupying lesions, the largest seen in segment V of the liver with ill-defined margins, mildly bright on T2 weighted images showing predominant peripheral enhancement suggestive of an early hepatic abscess. Smaller satellite lesions were also seen in the right lobe. Abnormal low T2 weighted signal intensity changes are seen in an infiltrative manner in the right lobe of the liver superior to the abscess with focal signal void areas (Figure 1). These findings were suggestive of hepatic abscesses in the right lobe with surrounding likely granulomatous hepatitis.

**Figure 2:**
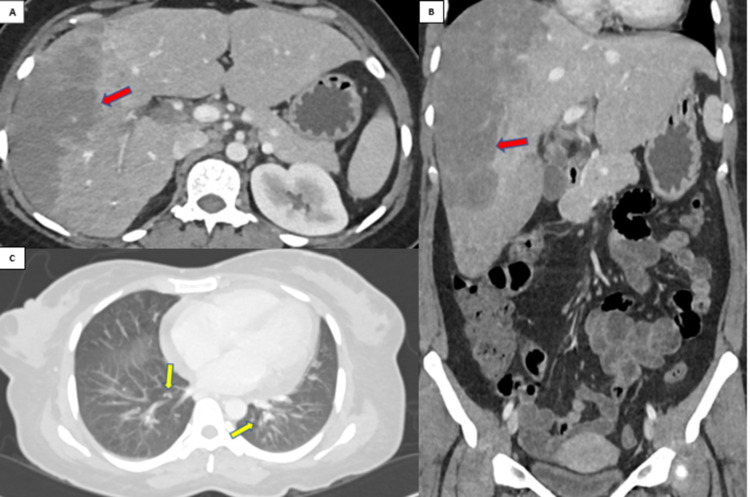
CT abdomen and pelvis with contrast, (A) axial and (B) coronal portal phase, showing enlarged liver with large ill-defined hypodense area in the right lobe (red arrows). (C) Axial lung window showing bilateral lung nodules.

Entamoeba serology came positive and the patient was started on intravenous metronidazole, paromomycin, and ceftriaxone. A CT thorax, abdomen, and pelvis were performed, and they found a large, ill-defined liver lesion that was non-enhancing and multiple satellite lesions in both lobes. Multiple chest nodules were seen bilaterally (Figure 2).

**Figure 3:**
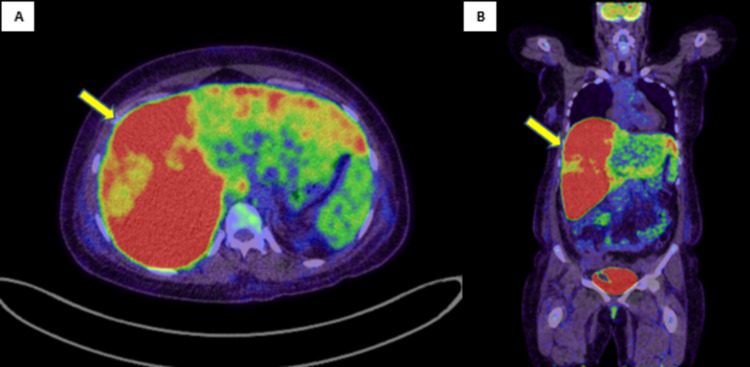
(A) Axial and (B) coronal PET CT showing intense diffuse uptake of FDG in the right lobe suggestive of diffuse involvement (yellow arrows).

Whole-body FDG PET (fluorodeoxyglucose positron emission tomography) CT showed intense diffuse uptake in the right lobe of the liver, suggestive of diffuse involvement with minimal involvement of the left lobe. Suspicious portocaval metastatic lymph nodes and lung nodes were also seen (Figure 3).

**Figure 4:**
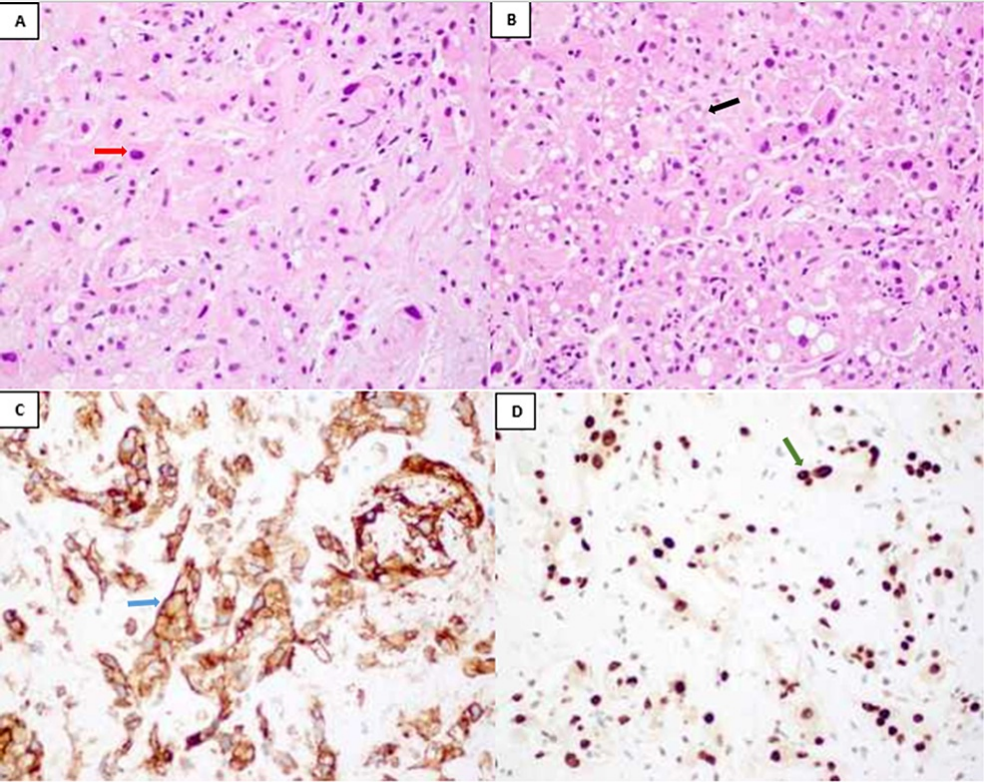
(A) Hepatic hemangioendothelioma composed of pleomorphic epithelioid cell (red arrow) with background myxohyaline stroma, (B) foci of tumor cells with prominent intracytoplasmic vacuoles (black arrow), (C) CD31 immunostaining highlighting the vascular nature of the lesional cells (blue arrow), and (D) ERG immunostaining demonstrating strong nuclear positivity (green arrow).

Liver biopsy showed pleomorphic epithelioid cells, with occasional intracytoplasmic vacuoles, seen within a myxohyaline stroma. The tumor cells were diffusely positive for vascular markers, namely CD31, CD34, and ERG (Figure 4). Cytokeratins and liver-specific markers such as HepPar1 and arginase 1 were negative. The morphology and immunohistochemistry were in keeping with a diagnosis of epithelioid hemangioendothelioma. Bone marrow (BM) aspirate was hypercellular with trilineage hematopoiesis and prominently increased erythroid precursors with severe dyserythropoiesis. No increase in blasts was noted. Viral serology, fungal culture, and tuberculosis tests were negative. Hb electrophoresis revealed the B-thalassemia trait.

The case was discussed in the hepatobiliary multidisciplinary team and the advice was for the right hepatectomy by the hepatobiliary surgeon. The patient started deteriorating on day 7, had multiple episodes of vomiting, and complained of severe pain in her right upper quadrant. Repeated blood investigations revealed increased CRP, prolactin, leukocytes with eosinophils, and thrombocytopenia. The patient further went into acute liver failure and hepatic encephalopathy. She was started on IV meropenem, tazocin, and steroids. She was shifted to the medical intensive care unit following a loss of consciousness. A CT head revealed no intracranial pathology. She went into disseminated intravascular coagulation and, unfortunately, could not survive.

## Discussion

According to the study by Mehrabi et al., the presentation of hepatic EHE is variable and can range from no symptoms at all to full-blown liver failure. Although a quarter of the patients were asymptomatic on presentation, the most common presenting symptoms for patients were weight loss, liver enlargement, and pain in the right upper quadrant of the abdomen. It was also noted that a significant proportion of the patients with hepatic EHE had some other organ involvement, with pulmonary metastasis leading the list [4]. Llueca et al. reported a patient presenting with a pelvic mass with liver metastatic lesions who was later found to have hepatic EHE [5], which shows how unpredictable the presentation of EHE can be.

EHE has a propensity to occur in the liver, lung, pleura, spleen, bone, brain, meninges, breast, stomach, lymph nodes, soft tissue, thyroid, prostate, and ovaries. It has been reported in more than 200 cases worldwide so far, and it most commonly occurs between the ages of 20 and 60 years old, with a predominance in females [2]. Hepatic EHE commonly presents with multilobar involvement with locally aggressive behavior. It is similar to angiosarcoma pathologically as a vascular origin tumor, however, with a relative improved prognosis. In most cases, it presents with right upper quadrant abdominal pain, hepatomegaly, and weight loss with metastasis commonly to the lung [4].

The main imaging features of hepatic EHE on magnetic resonance imaging (MRI) or computed tomography (CT) are the peripheral location of the nodules, the contraction of the capsule, and the tendency of multiple foci to merge [6,7]. It has been noted that the "lollipop sign" in which hypodense mass (the candy in the lollipop) on contrast with the termination of vascular stock (the stick) as it abuts the mass and the "target sign" on MRI, specifically on T2-weighted images, appears as a core with high intensity, a thin ring with low intensity, and a peripheral halo with a slight hyperintense signal [8,9]. Although these findings are specific to hepatic EHE, they can also be observed in hepatic abscesses, peripheral cholangiocarcinoma, and liver metastases. This makes the rapid diagnosis and management of patients challenging [10].

Imaging findings of hepatic EHE have been shown to be single, multiple, or diffuse nodular types [11]. On ultrasound imaging, the lesions appear hypoechoic when compared to the surrounding hepatic tissue, and on abdominal CT scan, lesions are usually reported as peripheral with extension to the capsule and capsular retraction that enhances peripherally in a nodular pattern after IV contrast administration [8]. On MRI images, T1-weighted lesions show low intensity relative to the surrounding liver, and on T2-weighted images, they appear heterogeneous with increased intensity. Some may have a targetoid appearance due to the presence of a central scar with peripheral increased cellularity. In contrast, a non-enhancing margin due to avascularity near the surrounding healthy liver parenchyma is noted. It has been suggested that ferumoxide-enhanced T1-weighted images characterize the tumor more in detail than other images [8].

The diagnosis of hepatic EHE is usually confirmed by pathological and immunohistochemical staining as the presentation and radiographic findings are usually nonspecific. The immunohistochemical staining usually shows positivity for vascular markers such as factor 8-related antigen, CD 31 and CD34 and is negative for carcinoma markers such as cytokeratins. Fli1 has been used as an ideal vascular marker for EHE [12, 13]. In addition, translocation of t(1;3)(p36.3;q25), resulting in the CAMTA1-WWTR1 fusion product, has been reported as the most commonly recognized genetic abnormality in this tumor [12]. This can also help to distinguish from the other differentials such as hepatic angiosarcoma, hepatocellular carcinoma, cholangiocarcinoma, and metastasis. Among these, angiosarcoma can be the most challenging as it is also positive for CD34 and CD31; hence, the use of a special stain such as CAMTA1 can be helpful to differentiate between the two [14]. However, in the majority of cases, this is not needed, as the morphological features of the two entities are usually distinctive.

As hepatic EHE is rare and no clinical trials are available, there is no standard treatment option at present and treatment can be variable, ranging from no treatment with observation to transplantation. However, the favorable treatment option for hepatic EHE is resection when it is a localized disease [15]. The option of chemotherapy is also not widely studied for hepatic EHE and the ultimate treatment is transplantation for tumors that are not resectable or that involve the larger area of the liver [16].

Prognosis of hepatic EHE can range from slowly progressive, similar to hepatic hemangioma, to a very aggressive disease like angiosarcoma [17]. The median survival of asymptomatic patients is over 10 years, while for advanced-stage patients it is as low as 75 months. This makes hepatic EHE a slow-progressing tumor [1,18]. In our case, the patient was treated as a case of a hepatic abscess, and later histopathology confirmed EHE in the liver with PAN CT revealing lung involvement as well. The late diagnosis made it difficult to manage and, unfortunately, the patient succumbed to death.

## Conclusions

Hepatic epithelioid hemangioendothelioma is a rare liver tumor with a variety of differential diagnoses, and early diagnosis and treatment significantly reduce mortality and morbidity. Appropriate evaluation includes imaging and biopsy. Treatment consists of surgical excision for localized disease and liver transplantation for extensive liver infiltration. Chemotherapy and ablation may also be considered for metastatic disease.

## References

[1] Kou K, Chen YG, Zhou JP, Sun XD, Sun DW, Li SX, Lv GY (2020). Hepatic epithelioid hemangioendothelioma: update on diagnosis and therapy. World J Clin Cases.

[2] Sardaro A, Bardoscia L, Petruzzelli MF, Portaluri M (2014). Epithelioid hemangioendothelioma: an overview and update on a rare vascular tumor. Oncol Rev.

[3] Sbaraglia M, Bellan E, Dei Tos AP (2021). The 2020 WHO Classification of Soft Tissue Tumours: news and perspectives. Pathologica.

[4] Mehrabi A, Kashfi A, Fonouni H (2006). Primary malignant hepatic epithelioid hemangioendothelioma: a comprehensive review of the literature with emphasis on the surgical therapy. Cancer.

[5] Alomari AI (2006). The lollipop sign: a new cross-sectional sign of hepatic epithelioid hemangioendothelioma. Eur J Radiol.

[6] Llueca A, Piquer D, Maazouzi Y, Medina C, Delgado K, Serra A, Escrig J (2018). Hepatic epithelioid hemangioendothelioma: a great mimicker. Int J Surg Case Rep.

[7] Hsieh MS, Liang PC, Kao YC, Shun CT (2010). Hepatic epithelioid hemangioendothelioma in Taiwan: a clinicopathologic study of six cases in a single institution over a 15-year period. J Formos Med Assoc.

[8] Lyburn ID, Torreggiani WC, Harris AC (2003). Hepatic epithelioid hemangioendothelioma: sonographic, CT, and MR imaging appearances. AJR Am J Roentgenol.

[9] Zhou L, Cui MY, Xiong J (2015). Spectrum of appearances on CT and MRI of hepatic epithelioid hemangioendothelioma. BMC Gastroenterol.

[10] Gan LU, Chang R, Jin H, Yang LI (2016). Typical CT and MRI signs of hepatic epithelioid hemangioendothelioma. Oncol Lett.

[11] Mamone G, Miraglia R (2019). The "Target sign" and the "Lollipop sign" in hepatic epithelioid hemangioendothelioma. Abdom Radiol (NY).

[12] Studer LL, Selby DM (2018). Hepatic epithelioid hemangioendothelioma. Arch Pathol Lab Med.

[13] Gill R, O'Donnell RJ, Horvai A (2009). Utility of immunohistochemistry for endothelial markers in distinguishing epithelioid hemangioendothelioma from carcinoma metastatic to bone. Arch Pathol Lab Med.

[14] Taniai T, Onda S, Sato S, Shiba H, Sakamoto T, Yanaga K (2020). Hepatic epithelioid hemangioendothelioma: difficult differential diagnosis from angiosarcoma. Case Rep Gastroenterol.

[15] Cao L, Hong J, Zhou L, Ye Y, Liu Y, Yu J, Zheng S (2019). Selection of treatment for hepatic epithelioid hemangioendothelioma: a single-center experience. World J Surg Oncol.

[16] Virarkar M, Saleh M, Diab R, Taggart M, Bhargava P, Bhosale P (2020). Hepatic hemangioendothelioma: an update. World J Gastrointest Oncol.

[17] Weiss SW, Enzinger FM (1982). Epithelioid hemangioendothelioma: a vascular tumor often mistaken for a carcinoma. Cancer.

[18] Makhlouf HR, Ishak KG, Goodman ZD (1999). Epithelioid hemangioendothelioma of the liver: a clinicopathologic study of 137 cases. Cancer.

